# Adipocytes-Derived Extracellular Vesicle-miR-26b Promotes Apoptosis of Cumulus Cells and Induces Polycystic Ovary Syndrome

**DOI:** 10.3389/fendo.2021.789939

**Published:** 2022-02-11

**Authors:** Guannan Zhou, Yuanyuan Gu, Fangyue Zhou, Hongdao Zhang, Menglei Zhang, Ganrong Zhang, Ligang Wu, Keqin Hua, Jingxin Ding

**Affiliations:** ^1^ Department of Gynecology, The Obstetrics and Gynecology Hospital of Fudan University, Shanghai, China; ^2^ Shanghai Key Laboratory of Female Reproductive Endocrine Related Diseases, Shanghai, China; ^3^ Changning Maternity and Infant Health Hospital, East China Normal University, Shanghai, China; ^4^ State Key Laboratory of Molecular Biology, CAS Center for Excellence in Molecular Cell Science, Shanghai Institute of Biochemistry and Cell Biology, Chinese Academy of Sciences, Shanghai, China

**Keywords:** PCOS (polycystic ovary syndrome), adipocytes, extracellular vesicles (EVs), miRNA, cumulus cells (CCs)

## Abstract

**Background:**

Polycystic ovary syndrome (PCOS) is a refractory reproductive disease and also a kind of endocrine and metabolic disease. Adipocyte cells can produce a mass of extracellular vesicles and orchestrate the status of other types cells. The objective of this study was to determine the effects of adipocyte-derived extracellular vesicles-miR-26b on cumulus cells (CCs) and development of PCOS.

**Methods:**

The crosstalk mediated by extracellular vesicle-miR-26b between adipocytes and CCs was determined in CC cells co-cultured with mature adipocytes or incubated with extracellular vesicle isolated from mature adipocytes. CCK-8 assay and flow cytometry were conducted in CCs treated with or without extracellular vesicles; microRNA (miRNA) sequencing was conducted for clarifying the key molecular. Hormone levels and ovary ovulation ability were conducted with animal experiment.

**Results:**

The results revealed that miR-26b was upregulated in extracellular vesicles derived from mature adipocytes. Adipocyte-derived extracellular vesicles inhibited viability and promoted apoptosis in CCs *via* targeting JAG1. Furthermore, extracellular vesicles derived from mature adipocyte disrupted the ovary ovulation and impaired the hormone levels.

**Conclusions:**

These results identify a novel signaling pathway that adipocytes-derived extracellular vesicles-miR-26b promotes cell apoptosis in CCs and disrupted the ovary ovulation in the development of PCOS. The study indicates that adipose tissue-derived extracellular vesicles-miR-26b may play a key role in the PCOS and also provides insight into developing new therapeutic strategies for PCOS.

## Introduction

Polycystic ovary syndrome (PCOS) is a common reproductive disorder among female individuals at reproductive age with an estimated incidence of 7%–9% (1). As a kind of heterogeneous endocrine disorder, PCOS is reported to affect up to 20% reproductive-age women worldwide (2).

It is well acknowledged that PCOS is a complex disease involved in multisystem including reproductive, metabolic, and psychological features. PCOS is characterized by hyperandrogenism, obesity, insulin resistance, polycystic ovarian morphology (PCOM), and/or anovulation, which disturbs numerous women at period of duration (3). The major endocrine disruption is excessive androgen secretion or activity, and a large proportion of women also have abnormal insulin activity. Many body systems are affected in polycystic ovary syndrome, resulting in several health complications, including menstrual dysfunction, infertility (4), hirsutism, acne, obesity, and metabolic syndrome. It was reported that women with PCOS have an established increased risk of developing type 2 diabetes (5) and a still debated increased risk of cardiovascular disease (6). The cause of polycystic ovary syndrome is unknown, but studies suggest a strong genetic component that is affected by gestational environment, lifestyle factors, or both. Recent studies found that obesity is closely related to PCOS (7), and diverse molecules derived from adipocytes play vital role in the PCOS (8).

Previous research has shown that extracellular vesicles (EVs) exist in the adipose tissue and adipocytes (9–11). When shuttled from a donor cell to a recipient cell, EVs can transfer numerous biological contents (12) (including microRNAs, proteins, and mRNAs) in extracellular vesicles, while extracellular vesicles derived from diverse cells and organs. It is widely acknowledged that microRNAs exert diverse functions in extracellular vesicles as a kind of important contents (13). Some studies have reported that extracellular vesicle microRNAs (miRNAs) could promote apoptosis in ovary (14) and some other endocrine organs (15). However, there is little evidence that extracellular vesicles-derived adipocytes play roles in PCOS through extracellular vesicle microRNAs.

The aim of this study was to assess the vital role of extracellular vesicles derived from adipocytes in the progression of PCOS and the mechanisms of miRNA encapsulated in adipocytes extracellular vesicles in PCOS. The results could broaden our understanding about the role of adipocytes in PCOS and provide support for long-term monitoring and management.

## Materials and Methods

### Patients and Specimen

The present study was approved by the Ethics Committee of Obstetrics and Gynecologic Hospital of Fudan University. A total of five patients with PCOS and five control patients were admitted between February 2019 and February 2020 in the study. Both of the two groups were with obesity judged by the Body Mass Index (BMI). Totally, five patients with PCOS and five normal women were enrolled as experimental and control group, respectively; sera were collected from all the participants between the second and fifth day of the menstrual cycle, followed by the isolated extracellular vesicles miRNA sequencing. Patient’s oral and written consent was obtained in all cases.

### Cumulus Cells Isolation

Cumulus cells were collected by ovariohysterectomy as previously described (16, 17). In brief, isoflurane was used to anesthetize the mice. Then, the ovaries were collected by ovariohysterectomy, then moved to the laboratory within 30 min at 37°C in sterilized saline. The ovaries were washed three times with phosphate‐buffered saline (PBS); they were minced with a surgical blade in 4-(2-hydroxyethyl)-1-piperazineethanesulfonic acid)-buffered tissue culture medium‐199 (Invitrogen) supplemented with 2 mmol/L NaHCO_3_, 5 mg/ml bovine serum albumin (BSA), and 1% (v/v) penicillin-streptomycin (Invitrogen). Based on the criteria of morphological normality and homogeneity in the cytoplasm, COCs were classified. Then, selected COCs were denuded by vortexing in 0.1% (w/v) hyaluronidase to separate cumulus cells. The cumulus cells were cultured in Dulbecco’s modified Eagle’s medium (DMEM) medium (Thermo Fisher Scientific Life Sciences, MA, USA) containing 10% fetal bovine serum (FBS) (Gibco, Grand Island, NE) and 1% antibiotic–antimycotic.

### Adipocytes Induction and Identification

Pre-adipocytes 3T3-L1 were cultured in DMEM containing 10% FBS. Mature adipocytes 3T3-L1 were induced by adipogenic induction treatment for 14 days [briefly, 2 days after confluence (day 0). Cells were treated with differentiation medium (5 μg/ml insulin, 1 μM dexamethasone, and 0.5 mM 3-isobutyl-1-methylxanthine (IBMX)] in DMEM supplemented with 10% FBS (Millipore Sigma) until day 8) and identified using Oil Red O staining.

### Cell Viability and Proliferation Assays

Cell viability was measured using a Cell Counting Kit-8 (CCK-8; Dojindo Laboratories, Kumamoto, Japan). Cells were seeded in 96-well plates (5 × 10^3^ cells/well) for 12 h, then treated with culture supernatant from pre-adipocytes/mature adipocytes or extracellular vesicles derived from pre-adipocytes/mature adipocytes for 12 h, followed by addition of 10 μl CCK-8 reagent per 100 μl culture medium. Cells were cultured for an additional hour and then measured at 450 nm using a spectrophotometer.

### Flow Cytometry Assays

Cumulus cells (CCs) were seeded in six-well plate (10^5^ cells/well) and cultured for 12 h. Cells were harvested 12 h after being co-cultured with pre-adipocytes/mature adipocytes or extracellular vesicles derived from pre-adipocytes/mature adipocytes. All the cells after treatment were collected and were detected by flow cytometry with the PE/Annexin-V Apoptosis Kit I (BD Biosciences, USA).

### Western Blotting Assays

CC cells (co-cultured with extracellular vesicles) were harvested and lysed with radioimmunoprecipitation assay (RIPA) (Beyotime, Shanghai, China), supplemented with phenylmethyl sulfonyl fluoride (PMSF) and phosphotransferase inhibitor (Beyotime, Shanghai, China). Protein concentration was determined with a bicinchoninic acid (BCA) kit (Beyotime, Shanghai, China), and then, a total of 30 μg of protein was loaded onto sodium dodecyl sulfate–polyacrylamide gel electrophoresis (SDS-PAGE) gels for electrophoresis and transferred to polyvinylidene fluoride (PVDF) membranes. The membranes were blocked in 5% fat-free milk for 12 h at 4°C and incubated in primary antibodies at 4°C overnight. Membranes were incubated with horseradish peroxidase (HRP)-conjugated secondary antibodies (1:1,000) (Cell Signaling Technology, Danvers, MA) for 1 h at room temperature and detected with Immobilon Western substrate (Millipore, Burlington, MA). Anti-JAG1 (ab7771) and anti-GAPDH (ab8245) antibodies were purchased from Abcam.

### Real-Time Fluorescence Quantification PCR Assays

The RNA extraction was conducted from isolated extracellular vesicles by an miRNeasy Micro Kit (Qiagen). Further miRNA expression analysis was conducted as previous reported. Briefly, 10 ng of transfer RNA (tRNA) was reverse transcribed with a miRCURY LNA RT Kit (#339340, Qiagen), and the housekeeping gene GAPDH was used for mRNA normalization and U6 used for miRNA normalization. The primer sequences used were listed as below: miR-26b forward, 5′- CGCCCTGTTCTCCATTACTT-3′ and miR-26b reverse, 5′- CCAGTGCAGGGTCCGAGGT-3′; GAPDH forward, 5′- ACAGTCAGCCGCATCTTCTT-3′ and GAPDH reverse, 5′- GACAAGCTTCCCGTTCTCAG-3′; and U6 forward, 5′- CGCTTCACGAATTTGCGTGTCAT-3′ and U6 reverse, 5′- GCTTCGGCAGCACATATACTAAAAT-3′. U6 was used for miRNA normalization; the results were analyzed by comparing 2^−ΔΔCT^ values.

### Extracellular Vesicles Isolation and Identification

First, we prepared extracellular vesicles-free FBS by depleting the bovine-derived extracellular vesicles *via* ultracentrifugation at 150,000 *g* at 4°C for 12 h. Then, we cultured the pre-adipocytes, and adipocytes were cultured in extracellular vesicles-free medium for 48 h, followed by the collecting conditioned medium. Then, we isolated and purified the extracellular vesicles by ultracentrifugation according to Thery’s protocol (18). Briefly, the collected conditioned medium was centrifuged at 300 g for 30 min to remove cells, and then, the supernatant was centrifuged at 2,000 *g* for 30 min to remove the cell debris. Next, the supernatants were ultracentrifuged at 120,000 *g* for 120 min at 4°C, and the pellet was collected as the extracellular vesicles. As for the characterization of extracellular vesicles, the morphology observation was conducted by transmission electron microscopy (FEI Tecnai G2 Spirit Twin, Philips, NL). The size measurement was conducted *via* nanoparticle tracking analysis (NTA) (Malvern, GB). The marker characterization was conducted *via* Western blot focusing CD63, Tsg101, and Alix. The quantification of extracellular vesicles was conducted depending on the protein measurement *via* the Bradford assay kit (New Cell & Molecular Biotech, Suzhou, CN).

### Extracellular Vesicles Internalization Assays

The internalization of extracellular vesicles into CC cells was observed by the laser confocal microscopy. Briefly, a pool of extracellular vesicles was labeled with PKH26 (Millipore Sigma) red fluorescent dye at the concentration of 2 μl/ml for 30 min at room temperature, and PKH26 labeled extracellular vesicles collection were conducted by ultracentrifugation at 100,000*g* at 4°C for 1 h. Then, the PKH26-labeled extracellular vesicles were resuspended in EV-free DMEM to co-culture with CC cells to assess their internalization (at the concentration of 5 × 103 extracellular vesicles/cell). Cells were fixed with 10% paraformaldehyde, and nuclei were stained with 4′,6-diamidino-2-phenylindole (DAPI) (Millipore) for 10 min at 4°C. Images were taken using a Leica DM200 fluorescent microscope and a TCS SP5 confocal laser scanning microscopy (Leica Microsystems, Wetzlar, GER). A total of 1 × 10^8^ extracellular vesicles were labeled with red fluorescent PKH26 dye (at the concentration of 2 μl/ml) for 30 min at 37°C and then washed and ultracentrifuged at 100,000*g* at 4°C for 1 h. As for the internalization of adipocytes-derived extracellular vesicles into ovary, the extracellular vesicles pellet (1 × 10^8^ extracellular vesicles/mice) was resuspended in phosphate-buffered saline (PBS) and injected intravenously into mice up to 12 h to detect internalization. After 12 h injection, ovaries were collected and fixed in 4% paraformaldehyde, followed by frozen section. Nuclei were stained with DAPI (Millipore) for 10 min. The internalization of adipocytes-derived extracellular vesicles into the ovary was evaluated using confocal microscopy.

### Animals and Experimental Protocols

The animal experiments were conducted in accordance with the criteria for the Care and Use of Laboratory Animals and approved by the Ethics Committee of Obstetrics and Gynecologic hospital of Fudan University. This study used 15 female C57BL/6 mice (7 weeks old, weighing 23.4 ± 1.6 g; Jackson Labs, Bar Harbor, WA), which were housed in a customized 12 h light/dark cycle at 24°C. Food and water were provided *ad libitum*. Mice were divided into three groups, including pre-adipocytes EVs injection, mature adipocytes EVs injection, and transfected miR-26b pre-adipocytes EVs injection groups. After 6 weeks of injection, the mice were killed, and the freshly dissected ovaries were subjected to subsequent experiments.

### High-Throughput Sequencing of MicroRNAs

The deep sequencing for microRNAs encapsulated in extracellular vesicles was conducted as described previously. Complementary DNA (cDNA) libraries were constructed with total RNA of extracellular vesicles derived from pre-adipocytes/mature adipocytes. Total RNA extraction was conducted directly from the isolated extracellular vesicles by the TRIzol Reagent (Ambion, USA). The RNA quantity measurement was conducted by the Qubit 2.0 Fluorometer (Invitrogen, USA). cDNA library construction (with 10 ng total RNA/each library) was conducted as previously described (18). The high-throughput sequencing of microRNA libraries was conducted by Illumina HiSeq 2000 sequencer.

### Luciferase Reporter Assays

Firstly, the prediction of the potential targets of miR-26b was conducted using the TargetScan (http://www.targetscan.org) and miRanda (http://miranda.org). Then, the fragments of the JAG1 3′UTR containing the wild-type (WT) or mutant (Mut) predicted binding site for miR-26b were subcloned into the pmirGLO (RiboBio). Then, CC cells were cultured and seeded in 24-well plates for further detection. Briefly, the miR-26b mimics or control (NC) sequences (RiboBio) were co-transfected with pmirGLO-JAG1 WT or pmirGLO-JAG1 Mut. After 48 h of transfection, cells were harvested and lysed. Next, firefly luciferase and Renilla luciferase substrates were added to measure the luciferase activity using the Dual-Glo Luciferase Reporter Assay System (Promega). The results of the luciferase assays were analyzed according to the manufacturer’s instructions.

### Extracellular Vesicles MicroRNAs Transfection Assays

To elucidate the function of specific miRNAs in extracellular vesicles derived from pre-adipocytes/mature adipocytes, miR-26b mimics and negative control (On-NC) (RiboBio, Guangzhou, China) were transfected into enriched 3T3-L1 derived extracellular vesicles by Lipofectamine 2000 Reagent (Invitrogen, USA). Briefly, we incubated the 3T3-L1-derived extracellular vesicles with 100 nM miRNA mimics in 100 μl KSFM supplemented with 2 μl Lipofectamine 2000 Reagent for 4 h. Then, the treated extracellular vesicles were washed with PBS and ultracentrifuged at 125,000 *g* for 1 h at 4°C. The pellet was collected as the mimic mir-containing extracellular vesicles.

### ELISA Assays

After 6 weeks of injection of adipocytes-derived extracellular vesicles, mouse blood samples collection was conducted. Briefly, the detection of the serum levels of luteinizing hormone (LH) and testosterone was conducted by the ELISA Kits (Yanhui, Shanghai, China).

### Statistical Analysis

Data were presented as means ± SEM. The statistical analyses were performed using SPSS 25.0 software (SPSS, Chicago, IL). The differences between groups were calculated using one-way ANOVA or Student’s t-test. Differences were considered statistically significant at p < 0.05.

## Results

### Adipocytes Inhibited CCs Growth Through Paracrine Route by Stimulating Apoptosis

**Figure 1:**
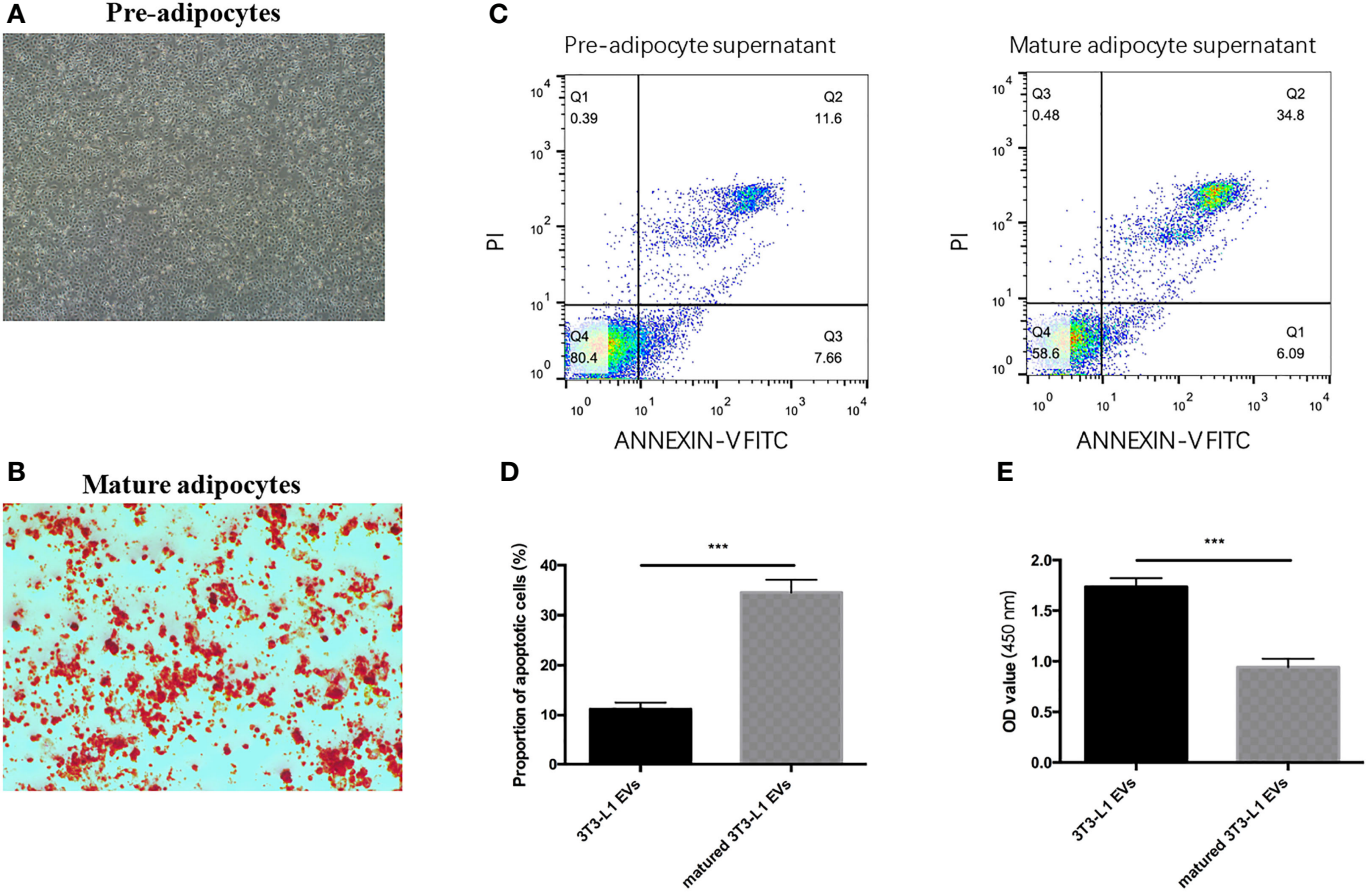
Adipocytes inhibited CCs growth through paracrine route by stimulating apoptosis. **(A)** Representative images of Oil red O staining of pre-adipocytes. **(B)** Representative images of Oil red O staining of mature adipocytes. **(C)** Detecting the apoptosis of CC cells induced by pre-adipocytes supernatant (left) and mature adipocytes supernatant (right) *via* flow cytometry. **(D)** Quantification of the apoptosis detected *via* flow cytometry. **(E)** Detecting the viability of CC cells when co-cultured with pre-adipocytes supernatant and mature adipocytes supernatant *via* CCK8 assay; ***p < 0.001.

It is known that adipose tissue has different biological functions in different stages of differentiation. The oil red staining assay showed that intracellular lipids accumulated in mature adipocytes but not in pre-adipocytes (**Figures 1A, B**). To confirm the paracrine effect of mature adipocytes in inhibiting cumulus cells proliferation, CCs were co-cultured with mature adipocytes, pre-adipocytes, and DMEM medium in the Transwell system. In the Transwell system, the cores only allowed molecules <0.4 μm to pass through and thus do not allow cells to pass through. When comparing the cell viability among diverse groups, cell viability in the mature adipocytes group was significantly inhibited (**Figure 1E**). In addition, mature adipocytes increased the percentage of apoptotic cells (**Figures 1C, D** **)**. These above results indicated that adipocytes inhibited CCs proliferation through secreted small molecules by stimulating cell apoptosis *in vitro*.

### Identification of Adipocytes-Derived Extracellular Vesicles

**Figure 2:**
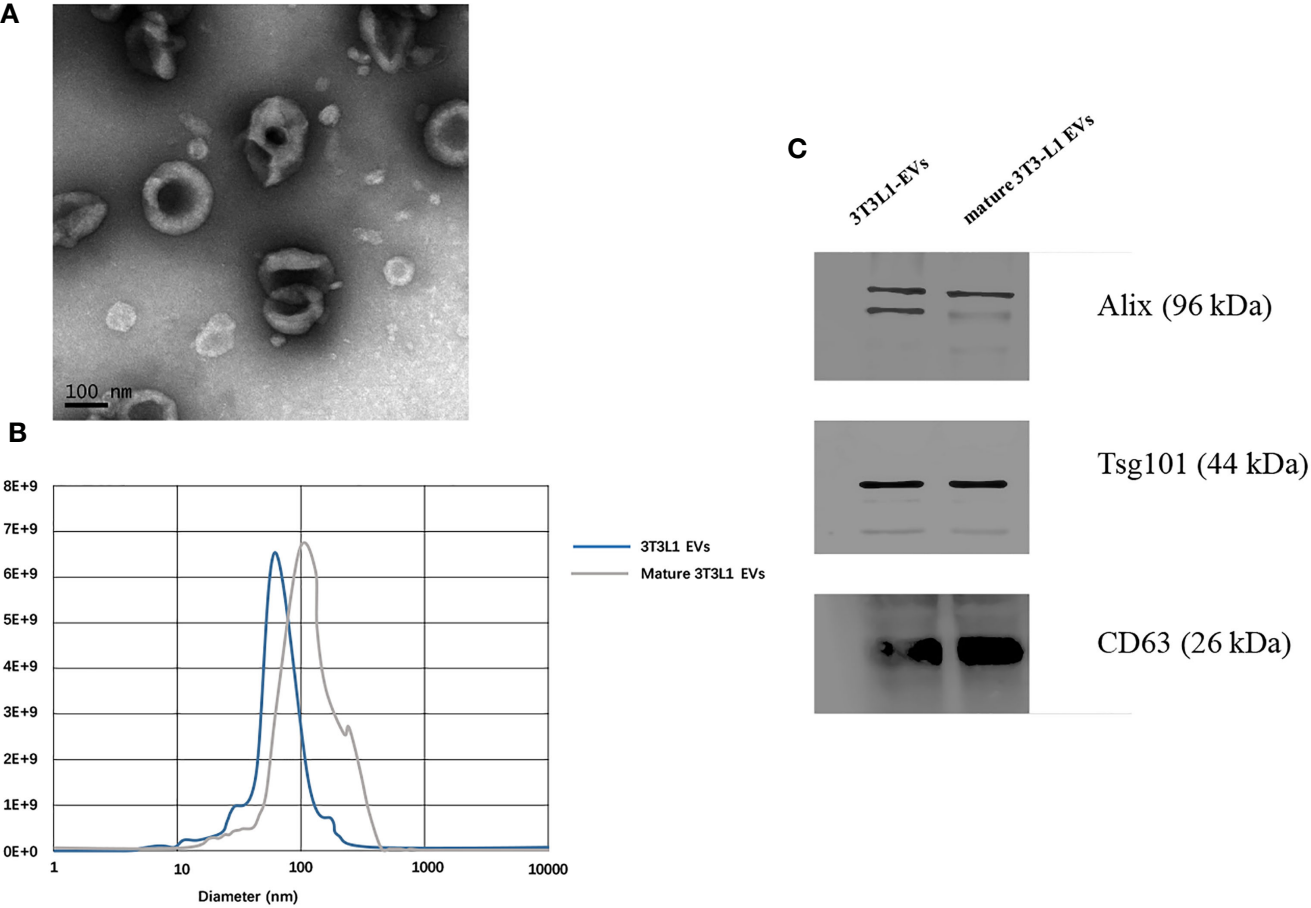
Identification of adipocytes derived extracellular vesicles. **(A)** Transmission electron microscope observation of whole-mounted purified extracellular vesicles derived from adipocytes. **(B)** Nanoparticle tracking analysis for particles size distribution of extracellular vesicles derived from pre-adipocytes/mature adipocytes. **(C)** Detection of CD63, Alix, and Tsg101 expression in extracellular vesicles derived from pre-adipocytes/mature adipocytes by Western blot.

Extracellular vesicles were isolated from cell medium supernatant *via* differential centrifugation. A transmission electron microscope illustrated the cup shape of adipocytes-derived extracellular vesicles (**Figure 2A**). Nanoparticle tracking analysis demonstrated that the distribution of the adipocytes-derived extracellular vesicles’ size mainly ranged from 30 to 200 nm (**Figure 2B**). In addition, the Western blot images showed that traditional extracellular vesicles markers including Alix, Tsg101, and CD63 were positively expressed in the extracellular vesicles derived from adipocytes (**Figure 2C** and **Supplementary Figure S1**). Overall, the above characterization results confirmed that the prepared isolation and collection were extracellular vesicles.

### Adipocytes-Derived Extracellular Vesicles Inhibited CCs Proliferation

**Figure 3:**
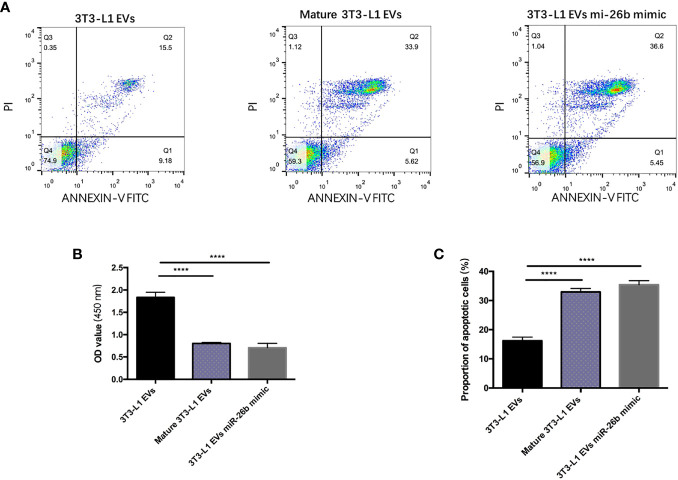
Adipocytes-derived extracellular vesicles inhibited CCs proliferation. **(A)** Detecting the apoptosis of CC cells treated with 3T3-L1 EVs (left), mature 3T3-L1 EVs (middle), and 3T3-L1 EVs mi-26b mimic (right) *via* flow cytometry. **(B)** Detecting the viability of CC cells treated with 3T3-L1 EVs, mature 3T3-L1 EVs, and 3T3-L1 EVs mi-26b mimic *via* CCK8 assay. **(C)** Quantification of the apoptosis detected *via* flow cytometry; ****p < 0.0001.

Cell apoptosis detection were conducted by flow cytometry analysis as previously described. After treatment with 1,000 ng/ml mature adipocytes-derived extracellular vesicles for 24 h, the viability of CC cells was inhibited when compared with the DMEM control and pre-adipocytes-derived extracellular vesicles (**Figures 3A, C**). Cell apoptosis assays indicated that mature adipocytes-derived extracellular vesicles increased the proportion of apoptotic cells in CC cells compared with pre-adipocytes-derived extracellular vesicles (**Figure 3B**). These results indicated that mature adipocytes promotes CCs apoptosis by the extracellular vesicles derived from them.

### Internalization of by CC Cells *In Vitro* and Ovary *In Vivo*

**Figure 4:**
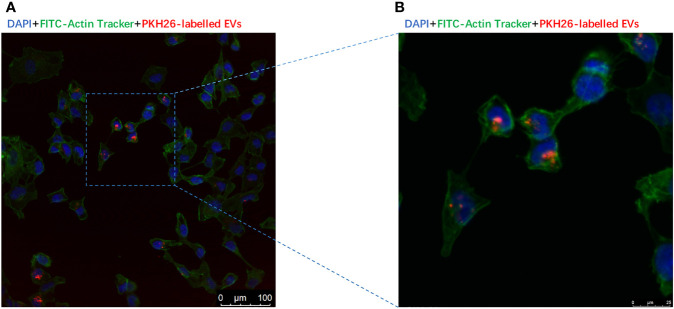
Extracellular vesicles derived from adipocytes were internalize by CC cells. **(A)** The presence of extracellular vesicles derived from adipocytes in CC cells was detected by confocal microscopy after incubating the PKH26-labeled EVs derived from adipocytes (red) with CCs. The nuclei of the CC cells were stained with DAPI (blue), and their cytoskeleton of the CC cells were stained with Actin-Tracker Green (green). **(B)** The magnification of extracellular vesicles derived from adipocytes internalized by CC cells.

**Figure 5:**
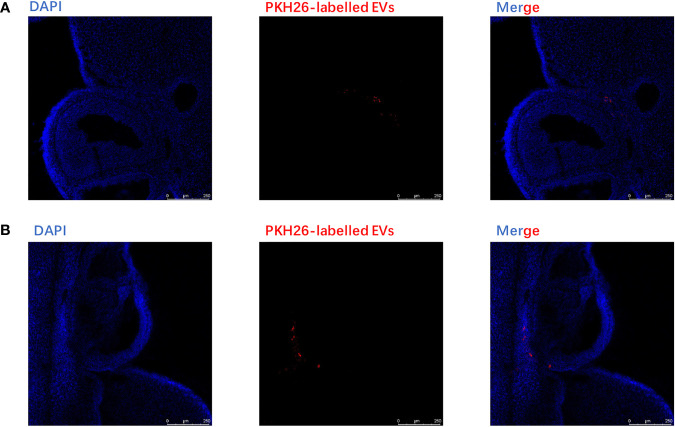
Extracellular vesicles derived from adipocytes were internalized by ovaries. **(A)** The analysis of extracellular vesicles derived from adipocytes in ovary was conducted by confocal microscopy after injecting the EVs intravenously in mice for 6 h. The PKH26-labeled EVs derived from adipocytes (red);, the nuclei of the CC cells were stained with DAPI (blue). **(B)** The analysis of extracellular vesicles derived from adipocytes in the ovary was conducted by confocal microscopy after injecting the EVs intravenously in mice for12 h. The PKH26-labeled EVs derived from adipocytes (red); the nuclei of the CC cells were stained with DAPI (blue).

PKH-26-labeled adipocytes-derived extracellular vesicles were treated with CC cells for 12 h. Laser confocal microscopy analysis showed that fluorescein isothiocyanate (FITC)-actin tracker labeled CC cells could internalize adipocytes-derived extracellular vesicles labeled with PKH26 (**Figures 4A, B**). Similarly, we injected PKH-26-labeled adipocytes-derived extracellular vesicles intravenously into mice for 12 h; after fixation and staining of the frozen section of resected ovaries, laser confocal scans showed that PKH26 red-labeled adipocytes-derived extracellular vesicle could enter into the ovary (**Figures 5A, B** **)**.

### Adipocytes-Derived Extracellular Vesicles Specific MicroRNAs Clarification and Function Prediction

**Figure 6:**
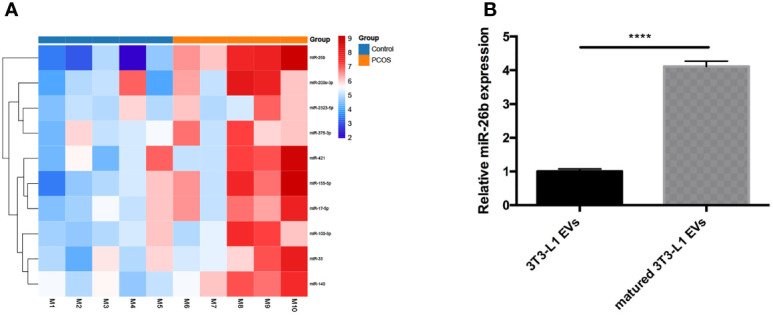
Analysis of differentially expressed extracellular vesicles miRNAs isolated from serum in diverse groups. **(A)** Heat map of differentially expressed miRNAs in the two groups. **(B)** Verification of miR-26b in the extracellular vesicles derived from pre-adipocytes and mature adipocytes; ****p < 0.0001.

To elucidate the contribution of miRNA of PCOS-patient-derived extracellular vesicles to PCOS development, miRNA sequencing analysis of PCOS samples and normal healthy samples was performed. We found that the miR-26b expression was remarkably increased in PCOS samples compared to healthy controls (**Figure 6A**). Increasing evidence support a vital role for miR-26b in modulating polymorphisms, obesity (19), insulin resistance (20), proliferation, apoptosis (21), tumorigenesis, invasion, migration, and angiogenesis; therefore, miR-26b was chosen for further validation and elucidation. In addition, we verified the results by using extracellular vesicles in granulosa cells of the PCOS mice and control mice; the result is in line with that of the sequencing results in patients (**Supplementary Figure S3**). To confirm these data, qPCR analysis was performed to determine the expression of miR-26b in the matured adipocytes-derived extracellular vesicles and pre-adipocytes-derived extracellular vesicles. The miR-26b expression was found to be increased in matured adipocytes-derived extracellular vesicles as compared to pre-adipocytes-derived extracellular vesicles (**Figure 6B**).

### miR-26b Promote CC Apoptosis by Regulating JAG1

**Figure 7:**
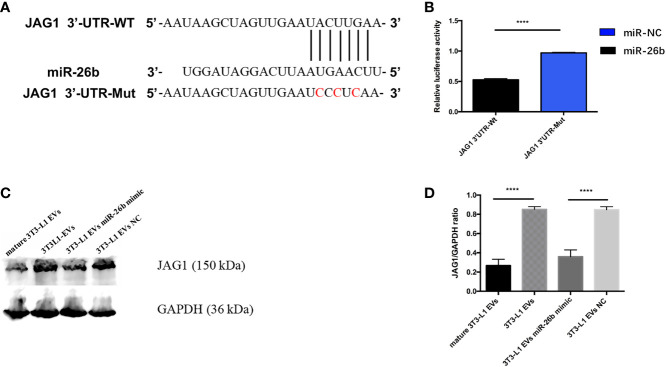
MiR-26b promote CC apoptosis by regulating JAG1. **(A)** Construction of the JAG1-3′-UTR WT and JAG1-3′-UTR Mut vectors and the active sequence of miR-26b. **(B)** Luciferase reporter assay was performed to detect the relative luciferase activities of WT and mutant JAG1 reporters. Upregulated miR-26b repressed the fluorescence intensity of JAG1-Wt-3′UTR. **(C)** Detecting JAG1 protein expression level after treatment with diverse extracellular vesicles by Western blot assay; treatment with both mature adipocytes- and pre-adipocytes-derived EVs miR-26b mimics significantly decreased the expression of JAG1 when compared with pre-adipocytes-derived EVs. **(D)** Quantification of the JAG1 expression when treated with diverse extracellular vesicles; ****p < 0.0001.

To further understand how miR-26b exerts its biological function, we used bioinformatics tools (TargetScan and microRNA.org) to predict the potential target of miR-26b. It was found that JAG1 possesses a targeting sequence for miR-26b at 3′ UTR of the gene. In addition, nucleotide mismatches between miR-26b and JAG1 were introduced in these binding regions of JAG1 through mutation (**Figure 7A**). The results from dual luciferase reporter assay showed that miR-26b negatively interacted with 3′ UTR of the wild-type JAG1, as seen from the decreased luciferase activity after miR-26b overexpression. In contrast, no interaction was seen between miR-26b and mutant JAG1 (**Figure 7B**). This evidence confirmed that JAG1 is directly inhibited by miR-26b. In addition, adipocytes-derived extracellular vesicles miR-26 mimic inhibited the viability (**Figure 3B**) of CC cells and increased the apoptosis (**Figure 3C**) when compared with pre-adipocytes-derived extracellular vesicles. In line with this, Western blot analysis indicated that cells that received extracellular vesicles containing overexpressed miR-26b demonstrated a decreased level of JAG1 (**Figures 7C, D** and **Supplementary Figure S2**).

### Adipocytes-Derived Extracellular Vesicles Regulated miR-26b Induced the PCOS in Mice

**Figure 8:**
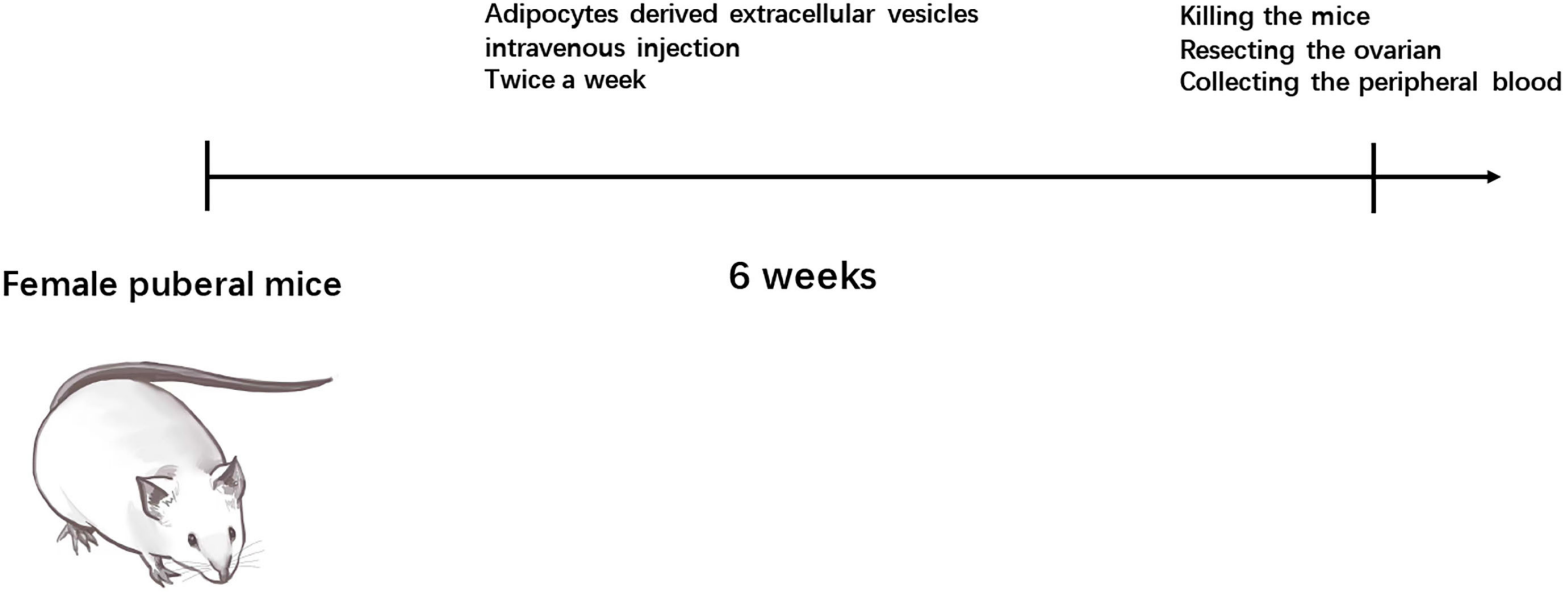
Timeline for the recipient mice treated with diverse EVs in promoting PCOS model.

**Figure 9:**
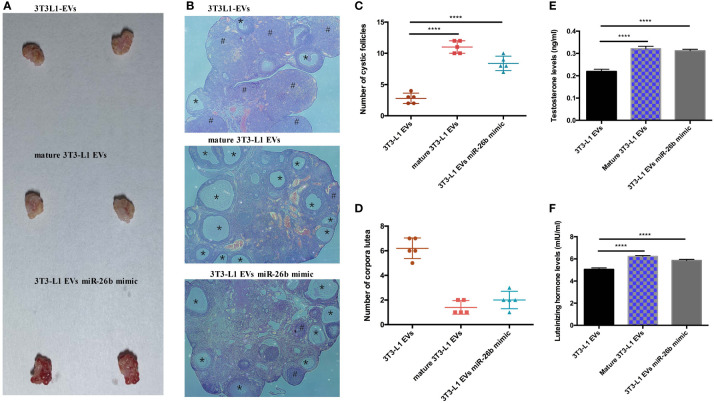
Adipocytes-derived extracellular vesicles regulated miR-26b induced the PCOS in mice. **(A)** Gross observation of ovaries when injected intravenously with 3T3-L1 extracellular vesicles (roof), mature 3T3-L1 extracellular vesicles (middle), and 3T3-L1 EVs miR-26b mimic (bottom). **(B)** Hematoxylin and eosin staining of representative ovaries; the cystic follicle is indicated by a # hashtag, while the corpora lutea are indicated by asterisks. **(C)** Quantitative analysis of cystic follicles. **(D)** Quantitative analysis of corpora lutea. **(E)** Quantitative analysis of testosterone levels. **(F)** Quantitative analysis of luteinizing hormone levels; ****p < 0.0001.

**Figure 10:**
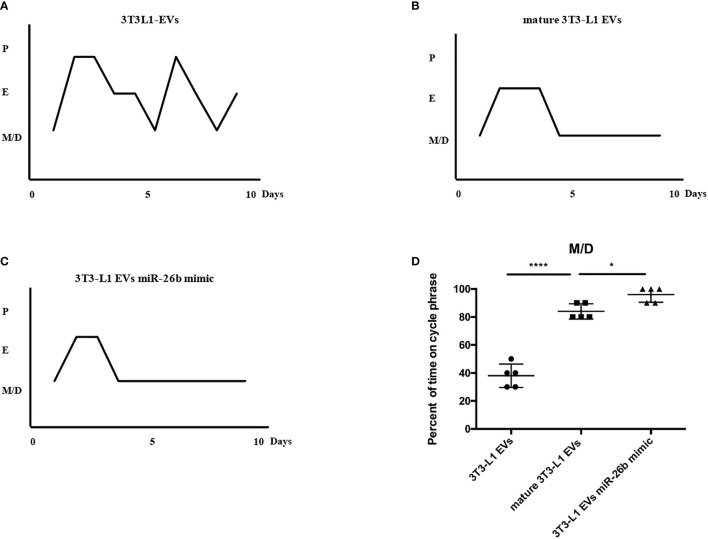
Representative estrous cycles for mice from the three groups. **(A)** Representative estrous cycles for mice treated after 3T3L1-EVs. **(B)** Representative estrous cycles for mice treated after mature 3T3L1-EVs. **(C)** Representative estrous cycles for mice treated after 3T3L1-EVs miR-26b mimic. **(D)** Quantitative analysis of estrous cycles for the mice from the three groups; *p < 0.05, ****p < 0.0001.

To explore the possible promotive effects of adipocytes-derived extracellular vesicles on PCOS in mice, extracellular vesicles derived from pre-adipocytes/mature adipocytes treatment impaired hormone levels and lead abnormal ovarian morphology. To investigate the effect of adipocytes-derived extracellular vesicles on the PCOS phenotype, extracellular vesicles from pre-adipocytes or mature adipocytes or transfected miR-26b were injected intravenously into mice for 6 weeks (**Figure 8**). After mature adipocytes-derived extracellular vesicles treatment, impaired ovarian morphologies were detected in the mature adipocytes-derived extracellular vesicles group and miR-26b mimic extracellular vesicles group (**Figures 9A, B** **)** but not in the pre-adipocytes-derived extracellular vesicles group, indicating that a rat PCOS model had been successfully developed *via* injection of mature adipocytes-derived extracellular vesicles or injection of miR-26b mimic extracellular vesicles. Ovaries from mice injected with mature 3T3L1 extracellular vesicles showed increased numbers of cyst-like follicles and fewer corpora lutea (**Figures 9C, D** **)**. In addition, dysfunctional hormone levels were also detected *via* ELISA assays (**Figures 9E, F** **)**. In addition, the estrus cycle results in **Figure 10** depict that the treatment of both mature adipocytes-derived extracellular vesicles group and miR-26b mimic extracellular vesicles disrupted the estrus cycle.

## Discussion

**Figure 11:**
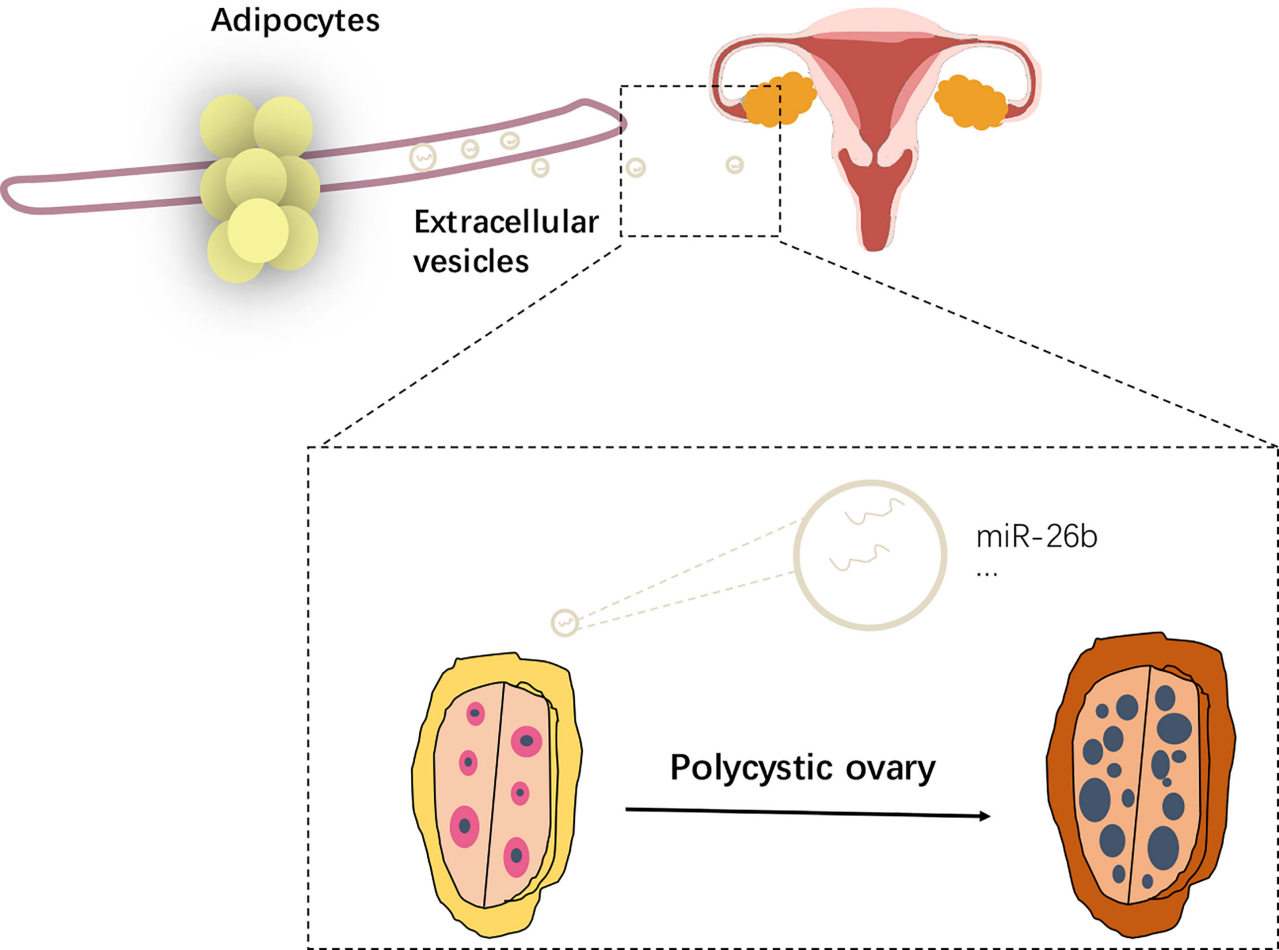
Schematic diagram showing the impact of extracellular vesicles derived from adipocytes in PCOS.

This study demonstrates that adipocyte‐derived extracellular vesicles are enriched with miR-26b and can be transported into CC in ovary not only *in vitro* but also *in vivo* (**Figure 11**). Matured adipocytes secrete miR-26b‐containing extracellular vesicles, which is internalized by CC cells and increase apoptotic rate. In the animal study, we conducted intravenous injection of extracellular vesicles derived from matured adipocytes into female mice and found that matured adipocytes-derived extracellular vesicles and miR-26b‐containing extracellular vesicles could disrupt the hormone levels and induced the PCOS in mice.

PCOS, acknowledged as one of the most common endocrine diseases, is characterized as ovulatory dysfunction, hyperandrogenism, and polycystic ovarian morphology (PCOM), which leads to substantial psychological, social, and economic burdens for patients and their couples and affects women at reproductive age (22). Obesity and ovulatory dysfunction (23) are major concerns of women with PCOS. Due to a limited understanding of the mechanisms involved, there is a limited demonstration about the mechanism between adipocytes and PCOS. Many studies demonstrated that PCOS ovarian phenotypes were related to obesity (23, 24). Adipose tissue is recognized as a main source of circulating miRNAs in the obesity status. MicroRNAs are acknowledged as the most important functional molecules in adipocytes extracellular vesicles (25, 26). There is wide variance in the abundance of different miRNAs between adipocytes- and pre-adipocytes derived extracellular vesicles (27, 28). However, whether adipocytes could induce the development of PCOS through extracellular vesicles is unclear yet. In this study, we first proposed that the mature adipocytes could inhibit the viability of CC cells and promote the apoptosis when compared with pre-adipocytes. Some studies believed that PCOS patients have the phenomenon of reduced apoptosis of granulosa cells (29), and the programmed apoptosis of granulosa cells is shown to be necessary for follicular maturation (30). However, in the current study, the apoptosis rate increased after treatment with adipocytes extracellular vesicles when compared with the control, which means that even if the programmed apoptosis of granulosa cells in the body is cycle specific, the apoptosis rate is increased when treated with the adipocytes extracellular vesicles. Furthermore, we found that the extracellular vesicles-derived mature adipocytes could be internalized by CC cells *in vitro* and by ovaries *in vivo*. The extracellular vesicles-derived mature adipocytes disrupt the normal secretory pattern, leading to the atresia of developing follicles and interference with the normal development of a dominant ovarian follicle, resulting in anovulation. In addition, the miRNA sequencing indicated that miR-26b is overexpressed in PCOS serum extracellular vesicles. It was reported that miR-26b plays a vital role in the aggravated insulin resistance and metabolic disorder in obesity. Extracellular vesicles could transfer miR-26b into CC cells and promote the apoptosis *via* targeting JAG1. What is more, when extracellular vesicles encapsulated with miR-26b mimics were injected intravenously into mice for 6 weeks, we found that extracellular vesicles could promote CC cells apoptosis *via* communicating with CC cells (transferring containing miR-26b into CC cells) and subsequently targeting JAG1 expression, which results in disrupted ovarian dysfunction associated with PCOS.

There are also some limitations in the presented study: the number of included PCOS cases is small, which might lead to the bias in searching the key microRNAs in the development of PCOS; also, the larger number of animals in the animal experiment is essential in the further study. In addition, the difference in the component of adipocyte extracellular vesicles between lean and obese PCOS patients is a valuable issue, limited by the size of the enrolled participants; this issue needs further exploring. What is more, due to the principles of ethics, it is difficult to conduct the experiments by using the follicular fluid in direct contact with granulosa cells or in granulosa cells of the PCOS women and control group. However, the results of the alternative assays we conducted (**Figure 7B**; **Supplementary Figure S3**) still make the results more convincing. Additional, the mechanism of extracellular vesicles derived from adipocytes among insulin resistance and PCOS urgently needs investigation.

All in all, our data indicate that the mechanisms underlying adipose-regulated ovarian dysfunction associated with PCOS likely involve adipose-derived extracellular vesicles containing miR-26b and directly affect ovarian granulosa cells to promote apoptosis.

## Data Availability Statement

The datasets presented in this study can be found in online repositories. The names of the repository/repositories and accession number(s) can be found below: BioProject ID: PRJNA784585.

## Ethics Statement

The studies involving human participants were reviewed and approved by the Ethics Committee of Obstetrics and Gynecologic hospital of Fudan University. The patients/participants provided their written informed consent to participate in this study.

## Author Contributions

GZ: Writing—original draft and editing. YG: writing—original draft. FZ: writing—review and editing and visualization. HZ: writing—review and editing. MZ: writing—review and editing. GRZ: writing—review and editing. LW: review and editing. KH: review and editing. JD: writing—review and editing, supervision, and funding acquisition. All authors contributed to the article and approved the submitted version.

## Funding

This work was supported by the National Natural Science Foundation of China (No. 81771524), National Natural Science Foundation of China (No. 91440107), and Natural Science Foundation of Shanghai (No. 21ZR1410400).

## Conflict of Interest

The authors declare that the research was conducted in the absence of any commercial or financial relationships that could be construed as a potential conflict of interest.

## Publisher’s Note

All claims expressed in this article are solely those of the authors and do not necessarily represent those of their affiliated organizations, or those of the publisher, the editors and the reviewers. Any product that may be evaluated in this article, or claim that may be made by its manufacturer, is not guaranteed or endorsed by the publisher.

## Supplementary Material

Supplementary Figure 1 Original image of western blot in **Figure 3C**.

Supplementary Figure 2 Original image of western blot in **Figure 8C**.

Supplementary Figure 3 Verification of miR-26b in the extracellular vesicles derived from granulosa cells of the PCOS mice and control mice; *P < 0.05, **P < 0.01, ***P < 0.001, ****P < 0.0001.
