# Supplementary figures and images for: Adipocytes-Derived Extracellular Vesicle-miR-26b Promotes Apoptosis of Cumulus Cells and Induces Polycystic Ovary Syndrome
Source: Front Endocrinol (Lausanne). 2022 Feb 11;12:789939. doi: 10.3389/fendo.2021.789939 (PMC8873091; doi:10.3389/fendo.2021.789939)

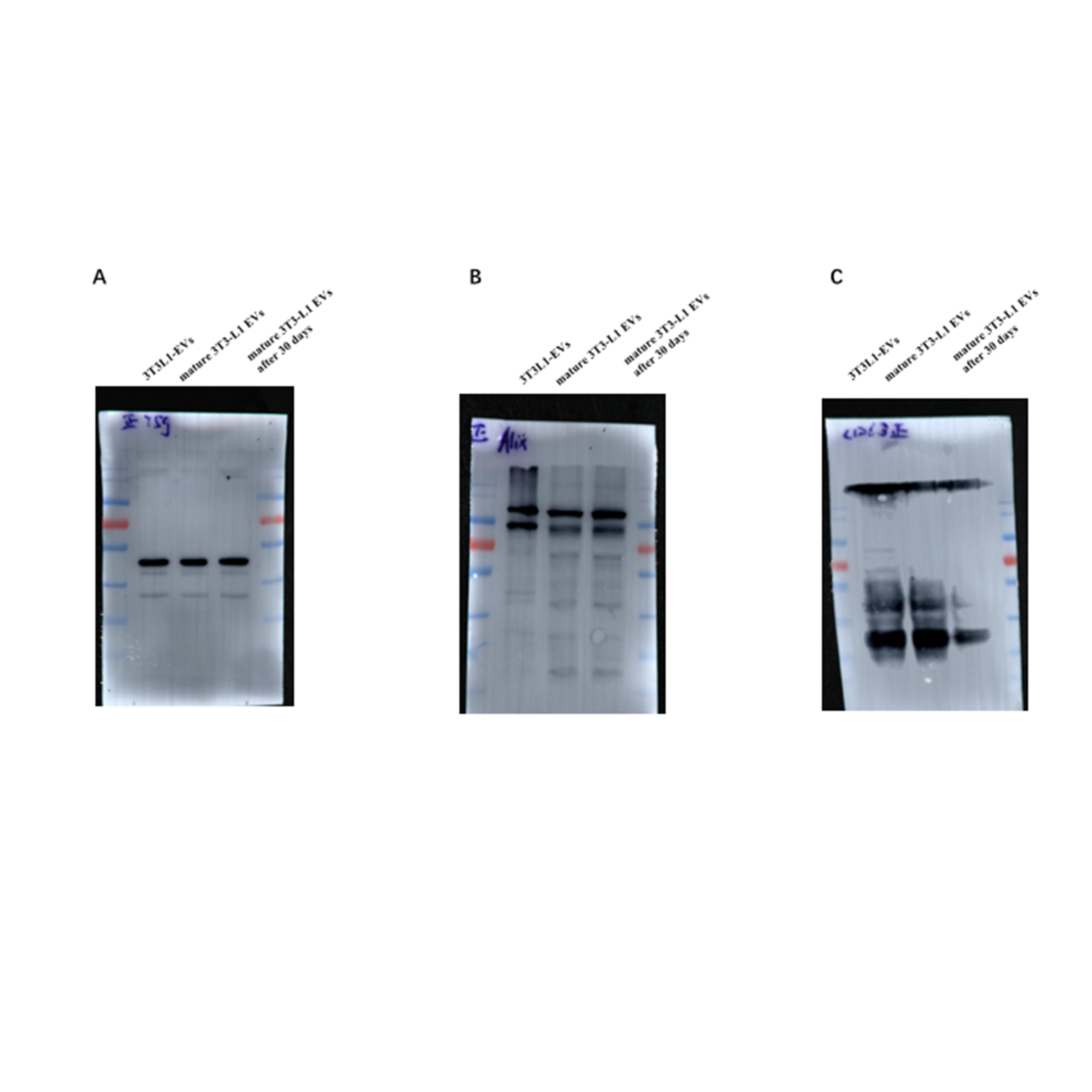

Supplement: Supplementary Figure 1 — Original image of western blot in Figure 3C . [file Image_1.tif]

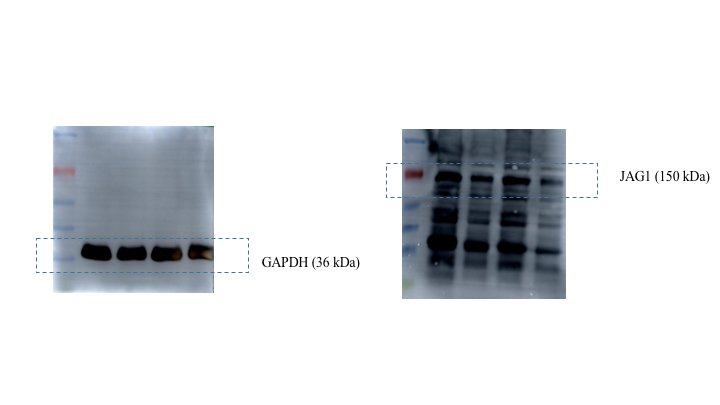

Supplement: Supplementary Figure 2 — Original image of western blot in Figure 8C . [file Image_2.tiff]

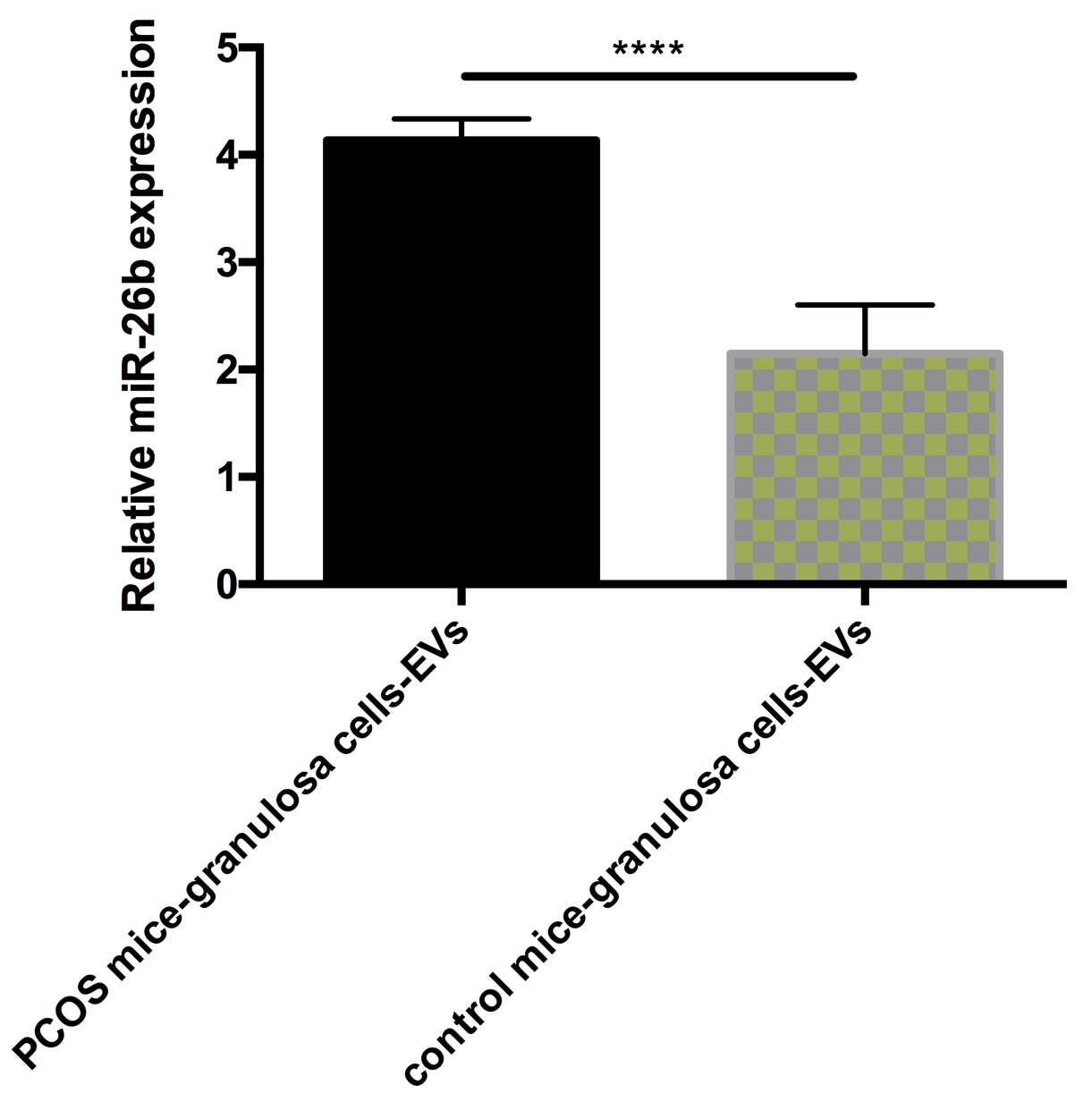

Supplement: Supplementary Figure 3 — Verification of miR-26b in the extracellular vesicles derived from granulosa cells of the PCOS mice and control mice; *P < 0.05, **P < 0.01, ***P < 0.001, ****P < 0.0001. [file Image_3.tiff]
